# Homemade ultrasound phantom for simulation of hydronephrosis

**DOI:** 10.1590/2175-8239-JBN-3916

**Published:** 2018-05-07

**Authors:** Ana Karine Brandao Novaes, Ramon Dalamura, Talita Menon, José Muniz Pazeli, Marcus Gomes Bastos

**Affiliations:** 1 Universidade Federal de Juiz de Fora, Juiz de Fora, MG, Brasil.; 2 Fundação Instituto Mineiro de Estudos e Pesquisas em Nefrologia, Juiz de Fora, MG, Brasil.; 3 Faculdade de Medicina de Barbacena, Barbacena, MG, Brasil.

**Keywords:** ultrasonography, hydronephrosis, models, educational, ultrassonografia, hidronefrose, modelo educacional

## Abstract

In this article, we describe the development of a simple and inexpensive simulation phantom as a surrogate of human hydronephrosis for the identification of urinary tract obstruction at bedside to be used in undergraduate training of medical students.

## Resumo

Neste artigo, descrevemos o desenvolvimento de um *phantom* de simulação, simples e barato, como modelo de hidronefrose humana para capacitar estudantes de graduação em medicina na identificação da obstrução do trato urinário à beira do leito.

## Introduction

Point-of-care ultrasonography (POCUS) done at bedside is a rapid, non-invasive, and safe procedure that allows clinicians to obtain timely vital information when examining patients, guiding several medical procedures.^1^ This initiative stimulated several medical schools in North America to introduce ultrasound (US) training for their undergraduate medical curriculum.^2^^-^ 5 Among the topics taught in these institutions, hydronephrosis, a result of an obstructed ureter, is listed among the 90 Medical Student Core Clinical Ultrasound Milestones, based on consensus from 32 medical education directors.^6^ However, the hands-on training in the US equipment is commonly done with students themselves as model, which does not allow the identification of abnormalities such as hydronephrosis in the training environment. Thus, the use of simulation phantoms present students with not only the possibility of obtaining competence in identifying pathologies such as renal pelvis dilatation by an obstructive process, but also allows the familiarization with the US machine in order to obtain images of good quality.^7^^-^ 11

In this paper, we describe a homemade, low cost, and quickly assembled phantom to be used as surrogate for training medical students and residents in the ultrasonographic identification of hydronephrosis.

## Methodology

**Figure 1:**
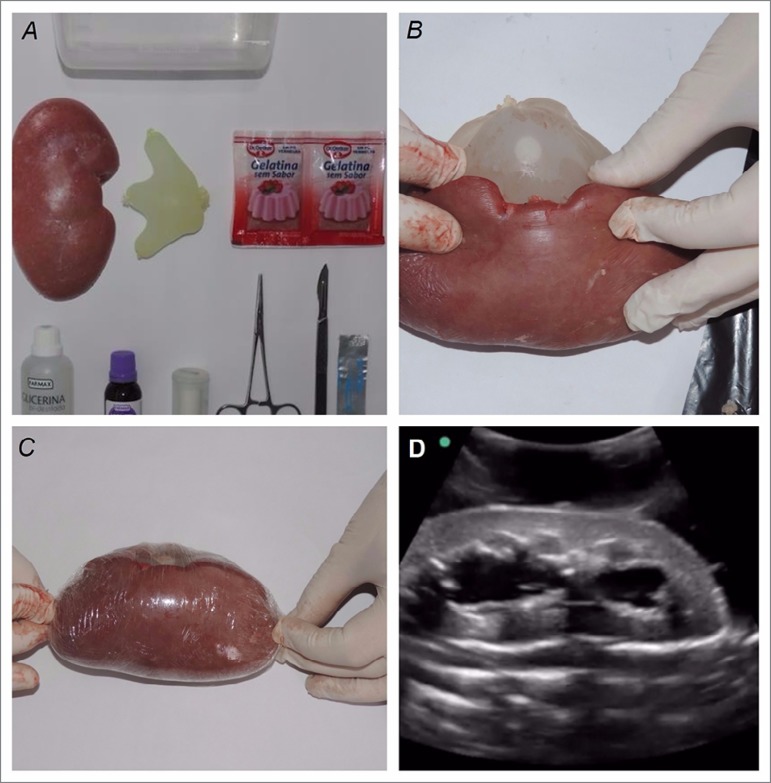
Assembling the hydronephrotic pork kidney

Based in previous publications describing techniques for creating gelatin based phantoms,^12^^-^ 14 we developed a distinct hydronephrotic renal structure, which allows the ultrasonographic differentiation of normal and obstructed kidney. The phantom was assembled using pork kidney, non-sterile gloves, dental floss, scalpel blade, bistoury, surgical tweezers, water, unflavored sugar-free gelatin, double distilled glycerin, 1% gentian violet, plastic film, kitchen oil, a plastic food container, oven, and refrigerator. The hydronephrosis was created using small size gloves with two of its fingers (first and fifth) previously tied and cut off. Water was then added to the glove, removing all the remaining air before tying the palm of the glove. Then, the water filled glove was carefully accommodated in a pouch previously made through an opening in the renal pelvis of a pork kidney, and kept in place by wrapping the kidney with plastic film. (Figure 1).

The tissue surrounding the kidney was simulated using melted gelatin (86 grams), glycerin (400 mL), and gentian violet (20 drops) to darken the mixture. In order to reproduce the depth of an adult human kidney, a base layer of the mixture was made in a plastic container previously greased with kitchen oil, and solidified in the refrigerator for approximately 3 hours. Next, the hydronephrotic kidney was placed over the gelatin base and the plastic container filled with enough gelatin mixture to cover the renal tissue with 3.5-4.0 cm, and chilled again in the refrigerator for 3 more hours. If kept in the refrigerator, the phantom can be used for about 3 to 4 weeks. The hydronephrosis phantom was then B-mode scanned using a curvilinear probe at 2-5 MHz attached to a SONOSITE M-TURBO machine.

This animal study was approved by The Commission of Ethics on the Use of Animals of the Pró-Reitoria of The Federal University of Juiz de Fora (protocol # 025/2017).

## Results

The immersed and nontransparent gelatin phantom made to simulate a hydronephrotic kidney allowed high quality and a quite comparable ultrasonographic image of an obstructed human kidney (Figure 1).

## Discussion

Over the last 25 years, POCUS has been used by general physicians to quickly obtain new information for the diagnostic process or to guide medical procedures. Ultrasound has been successfully included in several undergraduate medical curriculum as an extension of the physical examination; moreover, medical students find ultrasound training rewarding.^14^^-^ 15 While teaching normal anatomy can be done using students themselves as models, quite frequently appropriate human models with pathologies are not available in the training environment. Thus, appropriate training models that are life-like are needed during the hands-on teaching sessions.

In this study, we developed a hydronephrosis phantom made of pork kidney as surrogate of the human hydronephrotic kidney for teaching POCUS. The phantom described was assembled using easily accessible cheap materials (around US$ 25), and it replicates the ultrasonographic image of a human obstructed kidney. Although we did not made a formal evaluation of the phantom’s effectiveness in this article, it was used to test a group of medical students and residents to answer the question: In this ultrasonographic image obtained from a young man with flank pain, is hydronephrosis present?; all participants gave the right response. Another aspect of the phantom was the darkness of the gelatin obtained by adding gentian violet, which is important visualizing the kidney with the naked eye. Additionally, our phantom can be used to train nephrology and urology residents to improve their skills and confidence in US-guided percutaneous needle placement and, consequently, applying this skill in patient care.

In conclusion, we describe a hydronephrotic kidney made from pork kidney for simulation in hands-on practice of ultrasound. Its low cost plus quick and ease construction allows demonstrations of human hydronephrosis, a common clinical condition in emergency departments and recently identified among the Medical Students Core Clinical Milestones for undergraduate students.^6^
